# Numerical modeling the process of deep slab dehydration and magmatism

**DOI:** 10.1038/s41598-024-78193-w

**Published:** 2024-11-04

**Authors:** Hao Wu, Jiacheng Lei, Zeyu Jia, Jian Sheng, Yinan Zhu, Jian Wang

**Affiliations:** College of Transportation Engineering of Nanjing Tech, Nanjing Tech University, Nanjing, 211816 China

**Keywords:** Plate dehydration, Magmatic activity, Deep subduction zone, Mantle meltin, Magma chamber formation, Geology, Geophysics

## Abstract

This study uses a 2D high-resolution thermo-mechanical coupled model to investigate the dynamic processes of deep plate hydration, dehydration, and subsequent magmatic activity in ocean-continent subduction zones. We reveal the pathways and temporal evolution of water transport to the deep mantle during the subduction process. Plate dehydration plays a critical role in triggering partial melting of the deep mantle and related magmatic activity. Our study shows significant differences in the volumes of melt produced at different depths, with dehydration reactions in deeper regions being weaker compared to shallower ones. It takes a longer time to reach the suitable P-T conditions for hydrous melting in the deep mantle. The results highlight the geophysical significance of water transport in deep subduction zones and its role in magmatic processes, particularly in the formation of magma chambers beneath continental plates.

## Introduction

Water (as hydrogen) plays an important role in the geodynamics of our planet, not only by enhancing diffusion and creep^1,2^, but also by lowering the melting temperature of the rocks/mantle (i.e., ‘flux melting’)^3,4^. Furthermore, water can increase electrical conductivity^5^, significantly affect the solid-state viscosity of the convective mantle and dynamics of ascending plumes^6–8^, and trigger deep seismicity^9,10^. Slab dehydration significantly contributes to melt generation in the hot portion of the mantle wedge^3,4^and even in relatively cool environments above a subducting slab by lowering the melting temperature of rocks^6,11^. There is convincing evidence supporting slab dehydration at shallow and intermediate-depths (deeper crust and uppermost mantle)^12–14^. In contrast, the possibility and process of deep slab dehydration (at depth>300 km) are still poorly understood. However, deep slab dehydration is considered one of the most important dynamic processes in subduction zones because it provides a plausible explanation for the development of upwelling hydrous plumes^15^and intra-continental volcanism^16^, even though dehydration at such depths remains questionable.

Recently, water transport and melting have been considered in numerical modeling^17–21^, geological/geophysical observations^16,22^and laboratory experiments^23,24^. These studies reveal the mechanisms of deep water transport in subduction zones and their critical impact on the global water cycle.For example, Cai et al. (2018) revealed the depth distribution of plate hydration and the low-velocity zone in the upper mantle through three-dimensional seismic imaging in the central Mariana Trench^25^.Fujie et al. (2018) used ocean-bottom seismometers and reflection seismic data to study the Japan and Kuril trenches, discovering that plate bending faults play a crucial role in the hydration process. In particular, in the Japan Trench, the presence of faults significantly facilitates mantle hydration^26^.Grevemeyer et al. (2018) used global ocean-bottom seismic data to construct a velocity-depth model of the oceanic crust, investigating the impact of serpentinization on seismic characteristics, thereby revealing the complexity of the hydration processes within subduction zones^27^.Hermann and Lakey (2011) emphasized the decomposition process of water-rich chlorite-rich rocks under high-pressure conditions, highlighting the crucial role of these hydrous minerals in transporting water into the deep mantle in cold subduction zones^28^.Gies et al. (2024) used thermodynamic models of global subduction zones to assess the role of magnesium silicates and phase A in deep dehydration, revealing the importance of phase A’s dehydration reactions in the global water cycle^29^.

Laboratory studies further support these findings. Maurice, through high-pressure and high-temperature experiments, investigated the stability of hydrated magnesium silicates in subduction zones and found that phase E and phase A exhibit significant differences under varying conditions^30^.Howe and Pawley (2019) conducted an in-depth study on the stability of talc and the 10-Å phase under high-pressure conditions, revealing the influence of solid solution on mineral stability^31^.

Slab dehydration in subduction zones involves the decomposition of several hydrous phases, which can occur through either continuous or discontinuous reactions^23^. These reactions are pressure and temperature sensitive, with amphibole exhibiting a negative *dP/dT*slope^3,4,32–34^.Faccenda (2014) grouped the water processes in the subduction zone into three categories:

1. Hydration of dry oceanic lithosphere prior to subduction. In this stage, the oceanic slab stores water as seawater percolates down into the dry mafic and ultramafic rocks through faults and cracks, while magmatic activities contribute minor amounts to the water storage^35–37^.
2. The hydrated slab subducting into the mantle. In recent decades, seismic tomography and receiver functions have provided strong evidence for the presence of the hydrated slab in the deep mantle^22,38,39^.
3. Slab dehydration. The slab releases water by expelling pore fluid and loosely bound water (Physical dehydration) and by separating structurally bound water (Chemical dehydration) due to increasing pressure and temperature conditions during subduction^40,41^.

Shallow and intermediate-depth dehydration, which primarily releases pore fluids, has been evidenced by many geophysical observations, such as significantly lower electrical resistivity^14^, high *Vp/Vs*ratios associated with mainshock and aftershock sequences^13^, or the presence of a double seismicity zone (DSZ) in the mantle^12^. Serpentine (13 wt% H_2_O), chlorite (13 wt% H_2_O), talc (4.8 wt% H_2_O), brucite (31 wt% H_2_O) and amphibole (2.1 wt% H_2_O) are the primary hydrous phases in H2O-saturated peridotite at shallow depths (approximately 50 km) and under low-pressure conditions (*P*<2 GPa)23. At the depth between 50 and 150 km (2 GPa< *P*<5 GPa), chlorite becomes the dominant hydrous phase after serpentine decomposes. For moderate or hot slab geotherms, the deeper crust and uppermost mantle dehydrate most of water between 500 and 800 ℃^42^. For cold slab geotherms, fluids can be transported to greater depths (150 km< depth<300 km). Serpentine transforms into phase A (12 wt% H_2_O) at *P*>5 GPa, while serpentine, chlorite and talc transform into phase Å (13 wt% H_2_O) at 5 GPa< *P*<7 GPa and 600 ℃< T<700 ℃^27,29,43^. Laboratory experiments^24,44^have shown that the crust dehydrates completely at around 300 km, while significant hydration occurs in the upper mantle at intermediate depths in hot and almost all cold subduction zones (except for the coldest ones, such as the western Pacific)^41,45^.

However, systematic research on deep dehydration processes (beyond 300 km) and their impact on the mantle remains limited. Although some scholars believe that under high-temperature and high-pressure conditions, water is unlikely to penetrate deep into the mantle, increasing evidence suggests that subducting plates can transport water to depths exceeding 300 km. Furthermore, the release of deep water may trigger mantle melting and promote the upward migration of water, thereby influencing tectonic activity in subduction zones^46,47^. Stalder and Ulmer (2001) found that clinohumite (2.9 wt% H_2_O) can remain stable up to 1100 ℃ and 14 GPa^48^. This result implies that fluids can be transported down to transition zone (~ 440 km). Phase A and clinohumite transform into phase E (11.4 wt% H_2_O) at *P* >12 GPa, and as the pressure increases to 15–17 GPa, phase E transforms into superhydrous phase B (5.8 wt% H_2_O). The final phase of dense hydrous magnesium silicates (DHMS) is phase D (10–14 wt% H_2_O), which can remain stable down to 1200 km^49^. A series of solid-solid phase transformations in a cold slab geotherm, which can prevent slab dehydration, helps DHMS carry fluids down to the lower mantle43. Although DHMS has never been observed in nature and is unlikely to occur in the transition zone due to the high water solubility in wadsleyite and ringwoodite^50^, its presence makes it possible for fluids to be transported downward and/or released at depths greater than 300 km. There is still limited but growing evidence supporting slab dehydration at depths greater than 300 km^12,16^.

Therefore, this study aims to comprehensively examine the water migration process within subducting slabs throughout the entire subduction process using a numerical simulation model, with a particular focus on dynamic behavior in deeper regions.This study systematically investigates the dynamic process of deep dehydration in subducting slabs and its significant role in Earth’s deep processes using 2D high-resolution thermo-mechanical coupled oceanic-continental subduction models.We focus on dehydration events in subducting slabs at depths exceeding 300 km, as well as how the water released during these dehydration processes migrates within the mantle and influences partial mantle melting.

Through quantitative analysis, we estimated the amount of water released and its migration pathways under different geological conditions, and discussed the potential contribution of these dehydration events to magma activity and mantle plume formation.

## Methods

### Model setup

**Fig. 1:**
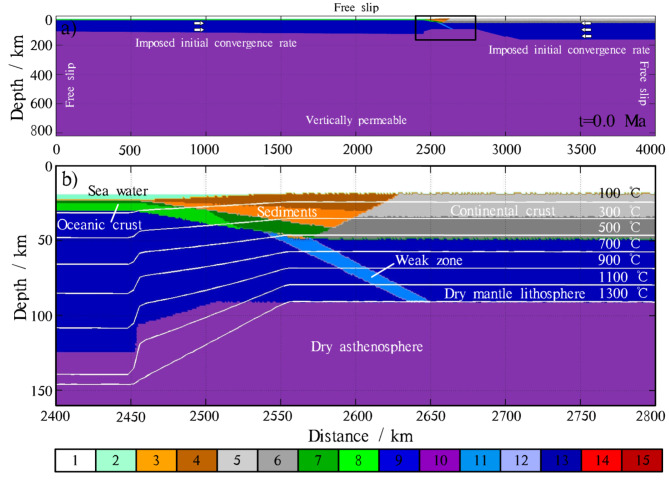
(**a**) Initial model geometry and boundary conditions (see text Sect. 2.1 of the text for details). The arrows on both sides indicate the locations where an initial convergence rate is imposed. (**b**) Enlarged composition field map of the trench area enclosed by the bold black line in (**a**). Isotherms are displayed as white lines with increments of 200 ℃, starting from 100 ℃. A weak zone, characterized by wet olivine rheology and low plastic strength (low coefficient of internal friction), is imposed between the subducting and overriding plates. The accretionary sediments are situated between the oceanic and continental crust, overlying the weak shear zone. The material legend is used for all model snapshots in this paper: 1-air, 2-sea water, 3-upper sediments, 4-lower sediments, 5-upper continental crust (felsic), 6-lower continental crust (felsic), 7-upper oceanic crust (basalt), 8-lower continental crust (gabbro), 9-dry mantle lithosphere, 10-dry asthenosphere, 11-weak initial shear zone, 12-hydrated mantle, 13-serpentinized mantle, 14-molten asthenosphere, 15-re-molten mantle.The map was generated using MATLAB (R2022a, MathWorks, https://www.mathworks.com).

Our model represents a cross-section of an ocean-continent subduction zone (Fig. 1), extending from the bottom of the upper mantle to the lithosphere, with an area of 820 km in depth and 4000 km in horizontal length. The model is based on the 2D thermo-mechanical code (I2ELVIS)^18,51^, which operates using finite differences and the marker-in-cell method. The 2D coupled thermo-mechanical model simulates the forced subduction of an oceanic plate beneath a continental plate. The model is divided into non-uniform rectangular grids with 2041 × 481 nodes, containing more than 10 million randomly distributed markers. The subduction zone (from *x* = 1498 km to *x* = 3002 km) has a high resolution (1 km×1 km) in both horizontal and vertical directions, while the rest of the region adopts a relatively lower resolution, which varies horizontally from 1.0421 km to 5.0046 km depending on the distance from the subduction zone. The initial set of materials is shown in Fig. 1, and detailed material properties are listed in Tables 1 and 2. A wet, low brittle/plastic strength, rheological olivine weak shear zone, with sin(*φ*) = 0.1 (where *φ* is the effective internal friction angle), is established between the overriding and subducting slabs to initiate subduction. The accretionary sediments are situated between the oceanic and continental crust, and overlie the weak shear zone (Fig. 1).

The bottom boundary of the model is open, while the other three boundaries are free-slip surfaces^52,53^. A boundary push (imposed on both the subducting and overriding plates) at a pre-existing weak zone initiates the subduction, until the oceanic slab sinks approximately 100–150 km into the mantle, at which point the subduction system can transition into a self-sustaining process. The initial thermal field in the subducting plate is set according to an oceanic geotherm^54^ corresponding to a 40 Ma lithospheric cooling age20. The initial temperature of the overriding continental plate linearly increases from 0 ℃ at the surface to 1367 ℃ at the lithosphere-asthenosphere boundary, below which an adiabatic temperature gradient of 0.5 ℃/km is applied.

In this study, we conducted a systematic sensitivity analysis by adjusting several key parameters (such as convergence rate, viscosity, etc.) one at a time, with other conditions held constant. Despite extensive comparative testing, this paper presents results selected from multiple simulations based on the depth of research and practical application considerations. The chosen scenario most closely reflects realistic geological processes and provides the most comprehensive depiction of the actual subduction processes of the Eurasian and Pacific plates.

**Table 1:** Material parameters used in the model.

Material	ρ_0_, kg/m^3^	c_0_, MPa	sin(φ_dry_)	Q_L_, kJ/kg	H_*r*_, µW/ m^3^	Cp, J/kg·K	α, 1/K	β, 1/MPa	Flow law
Sediments, volcanics from sediments	2600 (solid) 2400 (molten)	10	0.15	300	2	1000	3 × 10^−5^	1 × 10^−5^	Wet quartzite
Upper continental crust	2700 (solid) 2400 (molten)	10	0.15	300	1	1000	3 × 10^−5^	1 × 10^−5^	Wet quartzite
Lower continental crust	2700 (solid) 2400 (molten)	10	0.15	380	1	1000	3 × 10^−5^	1 × 10^−5^	Wet quartzite
Upper oceanic crust (basalt)	3000 (solid) 2900 (molten)	10	0.1	380	0.25	1000	3 × 10^−5^	1 × 10^−5^	Wet quartzite
Lower oceanic crust (babbro)	3000 (solid) 2900 (molten)	10	0.6	380	0.25	1000	3 × 10^−5^	1 × 10^−5^	Plagioclase An_75_
Volcanics from wet molten and subducted basalts and gabbro	3000 (solid) 2900 (molten)	10	0.15	380	0.25	1000	3 × 10^−5^	1 × 10^−5^	Wet quartzite
Lithoshpere-asthenosphere dry mantle	3300 (solid) 2900 (molten)	10	0.6	400	0.022	1000	3 × 10^−5^	1 × 10^−5^	Dry olivine
Reference ^a^	1,2	3	3	1,2	1	1,2	1,2	1,2	3

^a^(1) DonaldL.Turcotte & JerrySchubert^54^; (2) Bittner. & Schmeling^55^; (3) Ranalli^56^.

**Table 2:** Temperature and pressure dependent parameters used in the model.

Material	κ, W/m·K (at Tκ, *P*)	Tsolidus, K (at *P*)	Tliquidus, K (at *P*)
Sediments, volcanics from sediments	[0.64 + 807/(T + 77)]×exp(0.00004P)	889 + 17,900/(*P* + 54) + 20,200/(*P* + 54)2 at *P* < 1200 MPa. 831 + 0.06P at *P* >1200 MPa	1262 + 0.09P
Upper continental crust	[0.64 + 807/(T + 77)]×exp(0.00004P)	889 + 17,900/(*P* + 54) + 20,200/(*P* + 54)2 at *P* < 1200 MPa. 831 + 0.06P at *P* >1200 MPa	1262 + 0.09P
Lower continental crust	[0.64 + 807/(T + 77)]×exp(0.00004P)	973-70400/(*P* + 354) + 77,800,000/(*P* + 354)2 at *P* < 1600 MPa. 935 + 0.0035P + 0.0000062P2 at *P* >1600 MPa	1423 + 0.105P
Upper oceanic crust (basalt)	[1.18 + 474/(T + 77)]×exp(0.00004P)	973-70400/(*P* + 354) + 77,800,000/(*P* + 354)2 at *P* < 1600 MPa. 935 + 0.0035P + 0.0000062P2 at *P* >1600 MPa	1423 + 0.105P
Lower oceanic crust (gabbro)	[1.18 + 474/(T + 77)]×exp(0.00004P)	973-70400/(*P* + 354) + 77,800,000/(*P* + 354)2 at *P* < 1600 MPa. 935 + 0.0035P + 0.0000062P2 at *P* >1600 MPa	1423 + 0.105P
Volcanics from wet molten and subducted basalts and gabbro	[1.18 + 474/(T + 77)]×exp(0.00004P)	973-70400/(*P* + 354) + 77,800,000/(*P* + 354)2 at *P* < 1600 MPa. 935 + 0.0035P + 0.0000062P2 at *P* >1600 MPa	1423 + 0.105P
Lithoshpere-asthenosphere dry mantle	[0.73 + 1293/(T + 77)]×exp(0.00004P)	273.15 + 1085.17 + 13.29P-0.051P2-43$\:{\text{X}}_{{\text{H}}_{2}\text{O}}^{0.75}\:$b	273.15 + 1475 + 8P-0.32 P2-43$\:{\text{X}}_{{\text{H}}_{2}\text{O}}^{0.75}\:$b
Reference a	1,2	3,4,5,6,7,8	3

^a^(1) Clauser. & Huenges^57^, (2) Hofmeister^58^, (3) Schmidt & Poli^23^, (4) Hess^59^, (5) Hirschmann^60^, (6) Johannes^61^, (7) Poli & Schmidt^62^, (8) Katz et al^63^.. ^b^$\:{\text{X}}_{{\text{H}}_{2}\text{O}}^{0.75}$is the weight% of water Katz et al^63^.

### Hydration and dehydration process

**Fig. 2:**
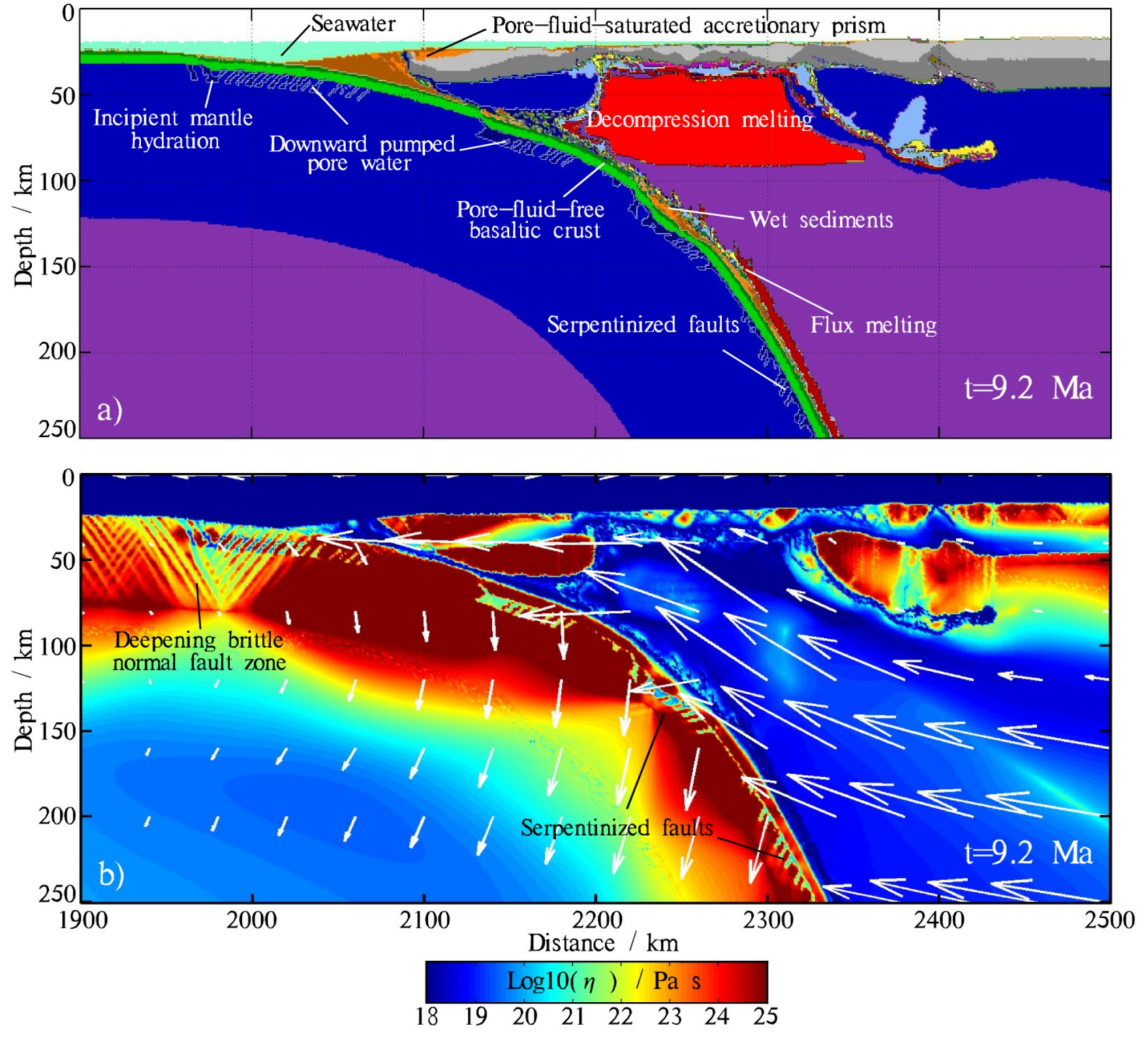
Snapshots of (**a**) the composition and (**b**) the viscosity fields of the reference model. White arrows in the viscosity snapshot represent velocities. Serpentinized faults are trenchward-dipping, but sets of antithetic and seaward-dipping faults are occasionally visible. The initial pore water in the basaltic layer is either pumped downward, leading to serpentinization, or expelled upward into the overlying mantle wedge and accretionary prism as the slab subducts. Fault activation due to the bending of the plate begins at shallow depths offshore of the trench and progressively deepens. The subducting slab becomes hydrated in the bending area as fluids percolate down into the dry mafic and ultramafic rocks through faults, cracks, or fluid infiltration. As the oceanic plate descends, the surrounding mantle begins to undergo flux melting. Decompression melting occurs beneath the back-arc extension zone.The map was generated using MATLAB (R2022a, MathWorks, https://www.mathworks.com).

Our model initially sets a homogeneously hydrated oceanic crust with a thickness of 7 km, composed of 2 km of hydrothermally altered hydrated basalts (4 wt% H_2_O) and 5 km of gabbros (1.4 wt% H_2_O). These water contents represent fluids, which primarily originate from the slab hydration process at the mid-ocean ridge. In our model, the main shallow hydration process occurs at the slab bending zone. Prior to subduction, the oceanic plate begins to bend downward, placing the near-surface rocks under tension, which leads to the generation of normal faults. Fluids percolate deeply into the bending oceanic plate along these normal faults, facilitating the downward migration of fluids and further reactivation^34^ (Fig. 2). Another shallow hydration process of the oceanic plate occurs when seawater percolates into the porous and permeable basaltic crust. The maximum serpentinization depth at the trench is set to reach down to sub-Moho levels (10–20 km). A finite brittle deformation threshold (e.g. $\varepsilon_{serp}$ =0.05 in our experiments) is set to control the extent of mantle serpentinization. When the deformation of the lithospheric mantle exceeds the prescribed threshold, the material transforms into serpentinized rock (2 wt% H_2_O) with 15% serpentinization (Fig. 2). The dataset for the initial maximum serpentinization is based on the average values estimated from tomographic studies at the trench^36,64,65^. At intermediate to deep depths, plastic behavior is not active. In our model, serpentinization of the slab interior occurs intrinsically, controlled by the reaction between excess fluids and the dry rocks through which they migrate.

Both porous compaction and dehydration reactions contribute to the expulsion of water from the subducting oceanic plate. Free fluids in hydrous basalts and gabbros gradually decrease from 4 wt% H_2_O/1.4 wt% H_2_O to zero at shallow depths (around75 km)^66^:

$$\:{\text{X}}_{{\text{H}}_{2}\text{O}}\left(\text{w}\text{t}.\text{\%}\right)=(1-0.013\cdot\:\varDelta\:\text{y})\cdot\:{\text{X}}_{{\text{H}}_{2}\text{O}}^{0}$$,

where $\:{\text{X}}_{{\text{H}}_{2}\text{O}}^{0}$ is the weight% of water at the surface, $\:\varDelta\:\text{y}$is the depth in km (0–75 km). We calculate the dehydration reactions/water release on the basis of physicochemical conditions and the assumption of thermodynamic equilibrium^20,66,67^. The hydration and dehydration processes are modeled using markers. A dehydration curve is used to control the dehydration reaction, which is defined as^23^:

$$\:\text{T}=478+0.18\times\:\text{P}-0.000031\times\:{\text{P}}^{2},\,at\,P<2.1\,GPa.$$

$$\:\:\:\:\:\:\:\:\text{T}=740-0.0018\times\:\text{P}-0.0000039\times\:{\text{P}}^{2},\,at\,P<2.1\,GPa.$$

where *T* is the dehydration temperature in ℃, and *P*is the current pressure in MPa. The released fluid is stored in a newly generated Lagrangian marker (referred to as a water marker) and propagates upward into the mantle wedge^51,68^. The migration velocity of the water marker is calculated as^53^:

$$\:{\text{v}}_{\text{x}}^{\text{w}\text{a}\text{t}\text{e}\text{r}}={\text{v}}_{\text{x}}^{\text{m}\text{a}\text{n}\text{t}\text{l}\text{e}},\:{\text{v}}_{\text{y}}^{\text{w}\text{a}\text{t}\text{e}\text{r}}={\text{v}}_{\text{y}}^{\text{m}\text{a}\text{n}\text{t}\text{l}\text{e}}-{\text{v}}_{\text{y}}^{\text{p}\text{e}\text{r}\text{c}\text{o}\text{l}\text{a}\text{t}\text{i}\text{o}\text{n}},$$

where $\:{\text{v}}_{\text{x}}^{\text{m}\text{a}\text{n}\text{t}\text{l}\text{e}}$ and $\:{\text{v}}_{\text{y}}^{\text{m}\text{a}\text{n}\text{t}\text{l}\text{e}}$ are the local velocities of the mantle in *x* and *y* direction, respectively. $\:{\text{v}}_{\text{y}}^{\text{p}\text{e}\text{r}\text{c}\text{o}\text{l}\text{a}\text{t}\text{i}\text{o}\text{n}}=10\:\text{c}\text{m}/\text{a}$describes the relative velocity of the upward percolation of water. The water marker propagates upward independently until it encounters mantle rocks, which can consume the water through hydration reactions or partial melting. The seismic data^69^ suggest that most mantle wedges contain average water contents of less than ~ 4 wt%, while saturated mantle rocks can contain up to ~ 8 wt% H_2_O^70^. Based on this observation, we assume an incompletely hydrated mantle wedge with 2 wt% H_2_O as a result of the channelization of slab-derived fluids^66,71,72^.

Studies have shown that deep slab dehydration in subduction zones is influenced by both slab morphology and pressure-temperature (P-T) conditions. These conditions can lead to regions with high water content and may trigger mantle melting^73,74^. Based on these findings, water migration during hydration and dehydration processes is primarily controlled by P-T conditions (see Table 2) and water stability equilibrium (≤ 2 wt% H2O), and is calculated based on geophysical-chemical conditions and thermodynamic equilibrium assumptions^20,66^.In our model, Perple_X is embedded as a function to control water migration through P-T conditions^70,75^. For example, the P-T conditions for the water content in sediments are set to P_max_= 7 GPa and T = 1500 °C, as sediments typically do not subduct into deeper parts of the mantle. In contrast, the P-T conditions for the water content in oceanic crust and mantle material are set to 30 GPa to simulate water migration throughout the entire upper mantle and the upper part of the lower mantle, including the mantle transition zone (MTZ)^76^.

During the calculation, the water content of each rock type is determined by tracking the P-T changes at each time step, with water migration is achieved through Lagrangian water advection points. These water advection points migrate upward independently until they are depleted by hydration or partial melting processes (0 wt% H2O)^51,53,77–80^.The equations governing the melting process in the model take into account pressure, temperature, water content, and the melting properties of minerals^63^.

### Magma-related process

Similar to the aforementioned hydration and dehydration processes, our experiments use Lagrangian Markers to track magma-related processes during model evolution (i.e. partial melting, melt extraction, percolation and accretion). A simplified method^81^ is adopted to implement magma-related processes in our 2D coupled thermal-mechanical models.

Katz et al^63^. presented a parameterization for melt fraction as a function of pressure, temperature, water content, and modal cpx, based on recent thermodynamic and experimental investigations. This melt fraction is applied in our models as:

$$\:{\text{M}}_{\text{c}\text{p}\text{x}}={\left(\frac{\text{T}-{\text{T}}_{\text{s}\text{o}\text{l}\text{i}\text{d}\text{u}\text{s}}}{{\text{T}}_{\text{l}\text{i}\text{q}\text{u}\text{i}\text{d}\text{u}\text{s}}^{\text{l}\text{h}\text{e}\text{r}\text{z}}-{\text{T}}_{\text{s}\text{o}\text{l}\text{i}\text{d}\text{u}\text{s}}}\right)}^{{{\upbeta\:}}_{\text{c}\text{p}\text{x}}}\:,\:\text{w}\text{h}\text{e}\text{n}\:{\text{T}}_{\text{s}\text{o}\text{l}\text{i}\text{d}\text{u}\text{s}}<\text{T}<{\text{T}}_{\text{c}\text{p}\text{x}-\text{o}\text{u}\text{t}}$$

$$\:{\text{M}}_{\text{o}\text{p}\text{x}}={\text{M}}_{\text{c}\text{p}\text{x}-\text{o}\text{u}\text{t}}+\left(1-{\text{M}}_{\text{c}\text{p}\text{x}-\text{o}\text{u}\text{t}}\right){\left(\frac{\text{T}-{\text{T}}_{\text{c}\text{p}\text{x}-\text{o}\text{u}\text{t}}}{{\text{T}}_{\text{l}\text{i}\text{q}\text{u}\text{i}\text{d}\text{u}\text{s}}-{\text{T}}_{\text{c}\text{p}\text{x}-\text{o}\text{u}\text{t}}}\right)}^{{{\upbeta\:}}_{\text{o}\text{p}\text{x}}}\:,\:\text{w}\text{h}\text{e}\text{n}\:{\text{T}}_{\text{c}\text{p}\text{x}-\text{o}\text{u}\text{t}}<\text{T}<{\text{T}}_{\text{l}\text{i}\text{q}\text{u}\text{i}\text{d}\text{u}\text{s}}$$

where $\:{\text{M}}_{\text{c}\text{p}\text{x}}$ is the degree of melting prior to the exhaustion of cpx, $\:{\text{M}}_{\text{o}\text{p}\text{x}}$ is the degree of melting for$\:\:{\text{T}}_{\text{c}\text{p}\text{x}-\text{o}\text{u}\text{t}}<\text{T}<{\text{T}}_{\text{l}\text{i}\text{q}\text{u}\text{i}\text{d}\text{u}\text{s}}$, and after the exhaustion of cpx, the melting reaction primarily consumes opx. T is the temperature in Kelvin, $\:{\text{T}}_{\text{s}\text{o}\text{l}\text{i}\text{d}\text{u}\text{s}}$ and $\:{\text{T}}_{\text{l}\text{i}\text{q}\text{u}\text{i}\text{d}\text{u}\text{s}}$ are the solidus and liquidus of the mantle, respectively (shown in Table 2). $\:{\text{T}}_{\text{l}\text{i}\text{q}\text{u}\text{i}\text{d}\text{u}\text{s}}^{\text{l}\text{h}\text{e}\text{r}\text{z}}$(lherzolite liquidus) is used to create a kinked melting function^63^. $\:{{\upbeta\:}}_{\text{c}\text{p}\text{x}}={{\upbeta\:}}_{\text{o}\text{p}\text{x}}=1.5$are the equation exponents derived from the “best fit” assemblage of the experimental solidus from Hirschmann^60^. $\:{\text{M}}_{\text{c}\text{p}\text{x}-\text{o}\text{u}\text{t}}$=0.15/(0.5 + 0.08*P*) (where *P* is the pressure in GPa) represents the total degree of cpx melting degree in a closed (batch) system. $\:{\text{T}}_{\text{c}\text{p}\text{x}-\text{o}\text{u}\text{t}}={\text{M}}_{\text{c}\text{p}\text{x}-\text{o}\text{u}\text{t}}^{{{\upbeta\:}}_{\text{c}\text{p}\text{x}}^{-1}}\left({\text{T}}_{\text{l}\text{i}\text{q}\text{u}\text{i}\text{d}\text{u}\text{s}}^{\text{l}\text{h}\text{e}\text{r}\text{z}}-{\text{T}}_{\text{s}\text{o}\text{l}\text{i}\text{d}\text{u}\text{s}}\right)+{\text{T}}_{\text{s}\text{o}\text{l}\text{i}\text{d}\text{u}\text{s}}$ is the temperature (in Kelvin) when cpx is exhausted through isobaric melting.

Partial melting is observed over a wide area in our model, which is similar to the melt pooling region^82^. The melt is extracted and stored in the shallowest part of the partial melting zone, and this process is tracked by Lagrangian markers in our models. The melt transportation is implemented by converting the markers (representing molten mantle/asthenosphere) into a new marker type (molten basalt) in the shallowest part of the melting pool (The shallow region where melt migrates upward and accumulates during partial melting). The total amount of extracted melt corresponds to the volume of the converted markers. The melt amount for each marker is calculated at every modeling time step using the following equation:

$$\:\text{M}={\text{M}}_{0}-\sum\:_{\text{i}=1}^{\text{n}}{\text{M}}_{\text{i}}^{\text{e}\text{x}\text{t}},$$

where$\:{\:\text{M}}_{0}$ is the standard melt fraction ($\:{\:\text{M}}_{0}={\text{M}}_{\text{c}\text{p}\text{x}},\:\:\text{w}\text{h}\text{e}\text{n}\:{\text{T}}_{\text{s}\text{o}\text{l}\text{i}\text{d}\text{u}\text{s}}<\text{T}<{\text{T}}_{\text{c}\text{p}\text{x}-\text{o}\text{u}\text{t}}$, $\:{\:\text{M}}_{0}={\text{M}}_{\text{o}\text{p}\text{x}},\:\text{w}\text{h}\text{e}\text{n}\:{\text{T}}_{\text{c}\text{p}\text{x}-\text{o}\text{u}\text{t}}<\text{T}<{\text{T}}_{\text{l}\text{i}\text{q}\text{u}\text{i}\text{d}\text{u}\text{s}}$), *n* indicates the number of previous extraction time steps, and $\:{\text{M}}_{\text{i}}^{\text{e}\text{x}\text{t}}$ represents the amount of extracted melt at the i-th time step. The rock is considered refractory/non-molten when the total extracted melt fraction ($\:\sum\:_{\text{i}=1}^{\text{n}}{\text{M}}_{\text{i}}^{\text{e}\text{x}\text{t}})$ is larger than the standard melt fraction ($\:{\text{M}}_{0}$). If the total amount of melt ($\:\text{M}$) exceeds a certain threshold, the melt in the marker is extracted, and $\:\sum\:_{\text{i}=1}^{\text{n}}{\text{M}}_{\text{i}}^{\text{e}\text{x}\text{t}}$is updated. The extracted melt is transmitted instantaneously to the emplacement area (e.g. the shallowest part of the melting pool where it forms a magma chamber), as the extracted melt leaves the melting zone much faster than rock deforms^83,84^. It is assumed that 80% of the extracted melt is emplaced at lower depths or beneath the continental plate, forming plutons in the continental crust in areas of highest possible intrusion emplacement. The remaining 20% can propagate upwards to the surface above the extraction zone, influencing surface topography evolution (e.g. forming volcanoes). A high potential local crustal divergence rate is used to predict the location of extracted melt intrusion emplacement, which is determined by the ratio of effective melt overpressure to the effective viscosity of the crust^66^:

$$\:{\text{d}\text{i}\text{v}}_{\text{c}\text{r}\text{u}\text{s}\text{t}}=\left[{\text{P}}_{\text{m}\text{e}\text{l}\text{t}}-\text{g}{\uprho\:}\left({\text{y}}_{\text{m}\text{e}\text{l}\text{t}}-\text{y}\right)-\text{P}\right]/{\upeta\:},$$

where $\:{\text{P}}_{\text{m}\text{e}\text{l}\text{t}}$ and $\:\text{P}$ are the pressures at the extraction depth ($\:{\text{y}}_{\text{m}\text{e}\text{l}\text{t}}$) and the current depth ($\:\text{y}$), respectively, $\:\text{g}$ is the gravitational acceleration, $\:{\uprho\:}$ is the melt density, and $\:{\upeta\:}$ is the current effective local crustal viscosity. Extracted melts are emplaced at the depth where the computed local crustal divergence rate is highest. To correctly couple the local and global flow field, the effects of matrix compaction in the melt extraction zone and crustal divergence in the melt emplacement area must be taken into account. We address these processes using a compressible continuity Eq. 68.

The effective values of other physical parameters of materials during magma-related processes are calculated using equations modified based on the melt fraction. The latent heating effect due to melting/crystallization equilibrium is accounted for by increasing the effective heat capacity and thermal expansion in the energy conservation equation:

$$\:{\text{X}}_{\text{e}\text{f}\text{f}}={\text{X}}_{\text{m}\text{o}\text{l}\text{t}\text{e}\text{n}}{\text{M}}_{\text{c}\text{p}\text{x}/\text{o}\text{p}\text{x}}+{\text{X}}_{\text{s}\text{o}\text{l}\text{i}\text{d}}(1-{\text{M}}_{\text{c}\text{p}\text{x}/\text{o}\text{p}\text{x}})\:$$

$$\:{\text{C}}_{\text{p},\text{e}\text{f}\text{f}}={\text{C}}_{\text{p}}+{\text{Q}}_{\text{L}}{\left(\frac{\partial\:{\text{M}}_{\text{c}\text{p}\text{x}/\text{o}\text{p}\text{x}}}{\partial\:\text{T}}\right)}_{\text{P}}$$

$$\:{{\upalpha\:}}_{\text{e}\text{f}\text{f}}={\upalpha\:}+{\uprho\:}\frac{{\text{Q}}_{\text{L}}}{\text{T}}{\left(\frac{\partial\:{\text{M}}_{\text{c}\text{p}\text{x}/\text{o}\text{p}\text{x}}}{\partial\:\text{P}}\right)}_{\text{T}}$$

where $\:{\text{X}}_{\text{e}\text{f}\text{f}}$ represents the effective value of a physical parameter, $\:{\text{X}}_{\text{m}\text{o}\text{l}\text{t}\text{e}\text{n}}$ and $\:{\text{X}}_{\text{s}\text{o}\text{l}\text{i}\text{d}}$ are the values of the physical parameter in the molten and solid phases, respectively. $\:{\text{Q}}_{\text{L}}$is the latent heat, and $\:{\text{M}}_{\text{c}\text{p}\text{x}/\text{o}\text{p}\text{x}}$ is the melt fraction ($\:{\text{M}}_{\text{c}\text{p}\text{x}/\text{o}\text{p}\text{x}}={\text{M}}_{\text{c}\text{p}\text{x}},\:\:\text{w}\text{h}\text{e}\text{n}\:{\text{T}}_{\text{s}\text{o}\text{l}\text{i}\text{d}\text{u}\text{s}}<\text{T}<{\text{T}}_{\text{c}\text{p}\text{x}-\text{o}\text{u}\text{t}},$ $\:\:{\text{M}}_{\text{c}\text{p}\text{x}/\text{o}\text{p}\text{x}}={\text{M}}_{\text{o}\text{p}\text{x}},\:\text{w}\text{h}\text{e}\text{n}\:{\text{T}}_{\text{c}\text{p}\text{x}-\text{o}\text{u}\text{t}}<\text{T}<{\text{T}}_{\text{l}\text{i}\text{q}\text{u}\text{i}\text{d}\text{u}\text{s}}$). All material parameters are listed in Tables 1 and 2.

## Results

We investigate the dynamic processes of slab hydration and dehydration in an ocean-continent convergence subduction system using 2D high-resolution thermo-mechanical models^51,68^. Particular attention is given to the evolution of deep slab dehydration and the transport of partial melts from deep magmatism.

### General model behavior

**Fig. 3:**
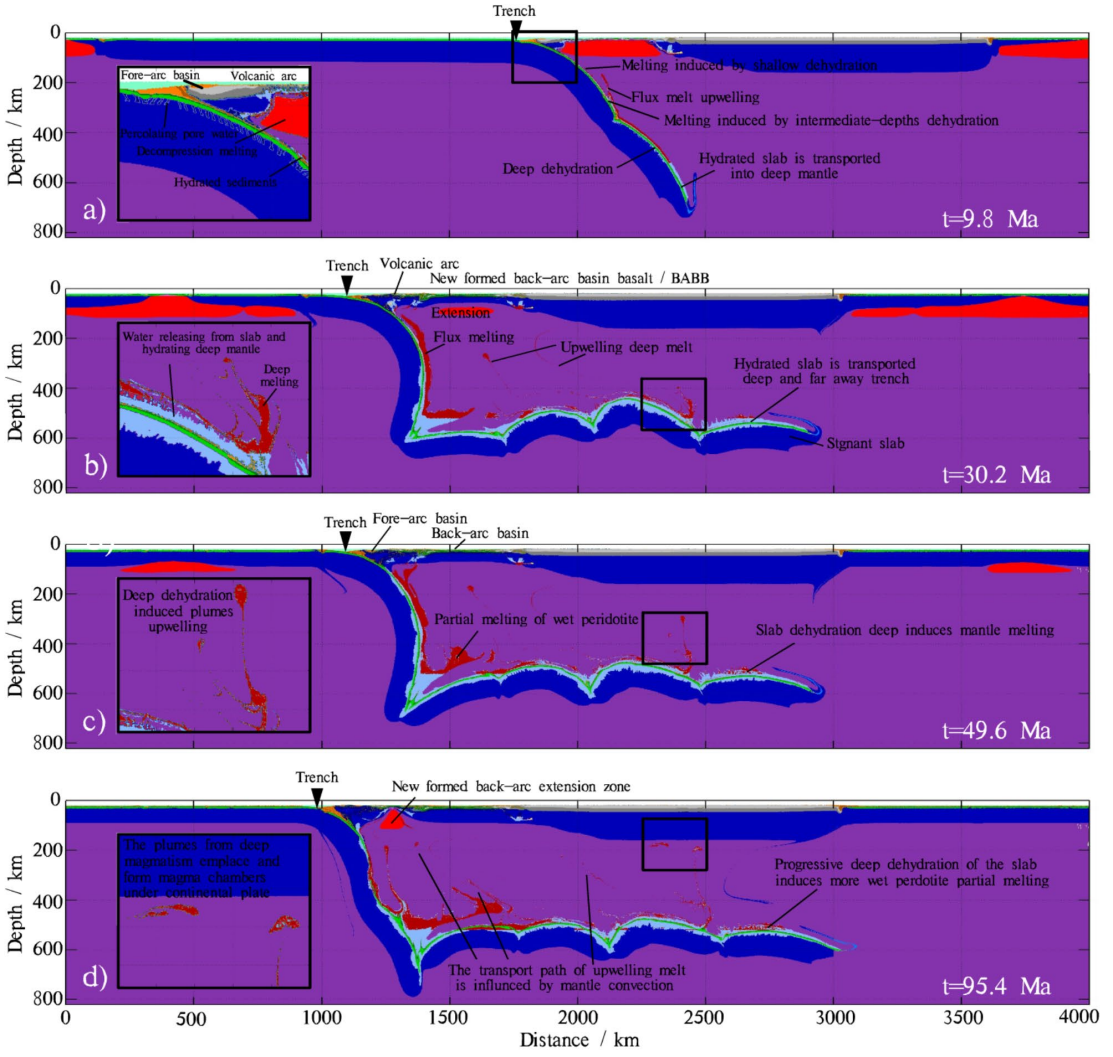
The general evolution of the reference model is illustrated by composition. The left inset figures in each snapshot are enlarged composition field maps, enclosed by bold black lines. (**a**) In the early stage of subduction, the oceanic slab becomes hydrated at the trench, and the hydrated slab is then transported into the deep mantle by subduction. (**b**) The stagnant slab transports fluids to deep regions far from the trench. These fluids are released and hydrate the surrounding mantle, leading to the generation of deep partial melts. At the surface, decompression melts and flux melts are extracted and emplaced beneath the back-arc extension zone, resulting in the formation of new back-arc basin basalts. (**c**) The stagnant slab is warmed by the surrounding hot mantle, inducing more water release and consequently generating more deep partial melts. d) The progressive deep dehydration of the stagnant slab induces more partial melting and upwelling of wet peridotite. The plumes generated by magmatism induced by deep slab dehydration are emplaced and form magma chambers beneath distant parts of the continental plate.The map was generated using MATLAB (R2022a, MathWorks, https://www.mathworks.com).

The general dynamic evolution of our reference model is shown in Fig. 3. In the earliest stage (Fig. 3a), the subducting slab becomes hydrated in the bending area, where seawater percolates down into dry mafic and ultramafic rocks through faults, cracks or fluid infiltration. As the oceanic plate descends, the surrounding mantle begins to melt due to the release of fluids from the hydrated surface of the subducting slab, which lowers the mantle solidus. Above the hydrated and partially molten mantle, a magmatic arc forms at the surface of the continental plate. The partially molten mantle rises upward, accompanied by the extension of the mantle lithosphere, leading to the formation of new volcanic crust at the surface. The hydrated slab is carried into the deep mantle by subduction.

The subducting slab reaches the 660 km transition zone, causing it to bend. The stagnant slab continues moving forward, transporting fluids to deep regions far from the trench (Fig. 3b). The deep portion of the subducting slab begins to release fluids, which hydrate the surrounding mantle and generate partial melts in the deep region. Some plumes resulting from deep slab dehydration rise upward. The migration path of these upwelling plumes is influenced by mantle convection. Furthermore, the decompression melts and flux melts are extracted and emplaced beneath the back-arc extension zone, leading to the formation of MORB-like back-arc basin basalts (BABB).

Subduction slows down due to a decrease in driving force and an increase in resisting force. The stagnant slab is warmed by the surrounding hot mantle. As a result, more water is released from the heated slab, leading to the generation of more deep partial melts. These deep partial melts coalesce into a mass and begin to upwell (Fig. 3c).

After a pause of several million years (e.g. 10 Ma), the oceanic plate resumes subducting. The trench begins to retreat again, and a new back-arc extension zone is formed. The episodicity of subduction has been observed in many subduction zones, such as the central Mediterranean region^85^, the Parece-Vela Basin, and the Mariana Trough^86^. During this period, the deep part of the subducting slab becomes hotter, generating more partial melts. The plumes generated by deep slab dehydration-induced magmatism are emplaced and form magma chambers beneath distant parts of the continental plate (Fig. 3d).

### Water transport

**Fig. 4:**
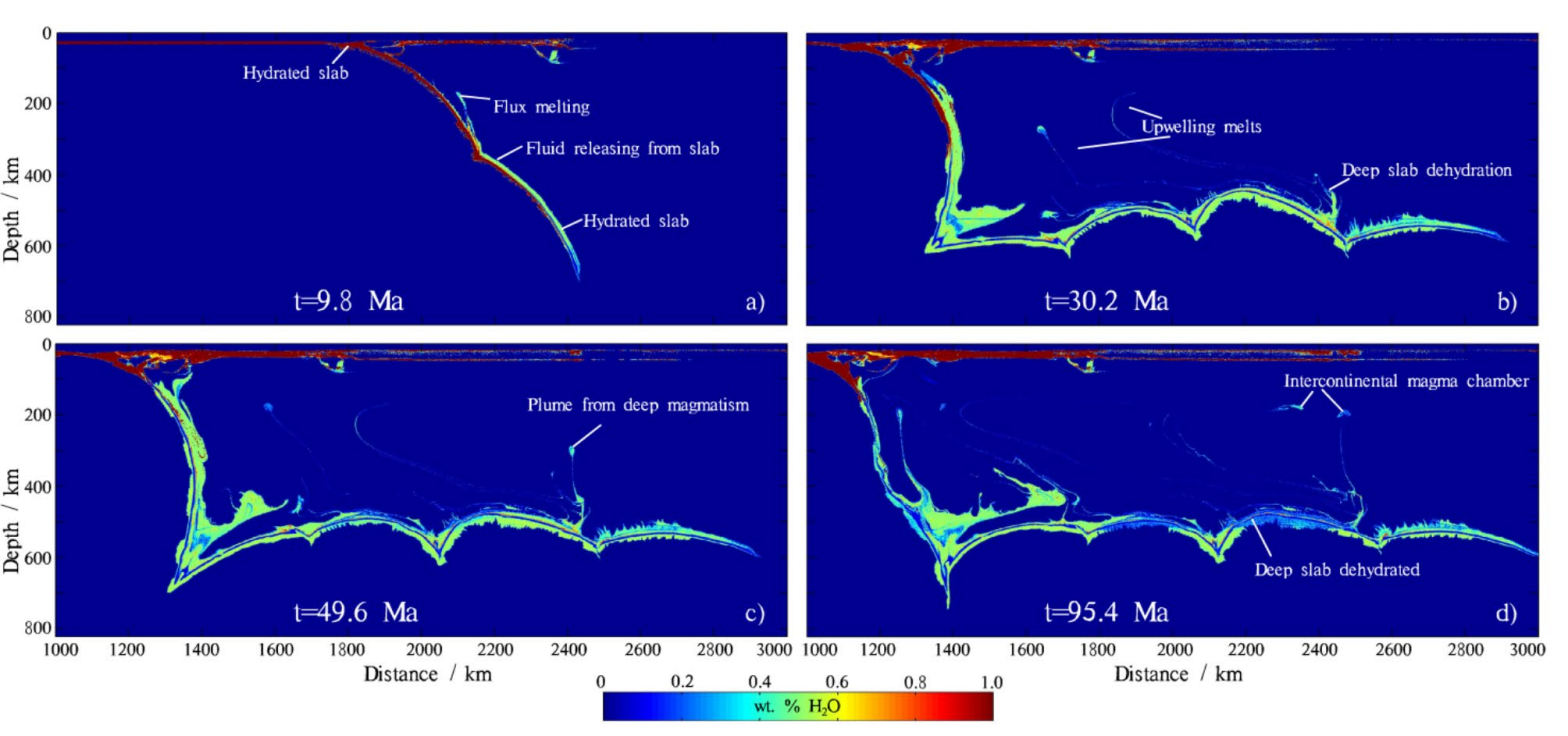
Color snapshots illustrate the general evolution of water content in the reference model. The water content is displayed in units of weight% (wt% H_2_O). a)-d) represent different stages of subduction.The map was generated using MATLAB (R2022a, MathWorks, https://www.mathworks.com).

Initially, the subducting slab becomes hydrated at the trench and carries more than 1 wt% H_2_O into the mantle (Fig. 4a). The fluids carried by the slab are gradually consumed by dehydration as subduction continues. These fluids are not fully exhausted at shallow and intermediate depths but are instead carried into the deep mantle by the subducting slab. A series of solid-solid phase transformations in a cold slab geotherm involving DHMS can prevent slab dehydration and help carry fluids down to the lower mantle^43^. Approximately 0.4–0.8 wt% H_2_O can be transported to the ~ 660 km transition zone (Fig. 4a). The stagnant slab, warmed by the surrounding hot mantle, releases additional fluids, which in turn hydrate the mantle above the slab. The hydrated mantle contains approximately 0.4–0.6 wt% H_2_O, and in some regions, the fluid content can reach up to ~ 0.9 wt% H_2_O (Fig. 4b). In some areas of the stagnant slab, where the released fluids cannot be completely consumed by hydration reactions or partial melting, the remaining fluids penetrate upward and hydrate the mantle wedge above the slab (Fig. 4c). If the volume of these fluids is sufficient, they can even transport 0.4–0.5 wt% H_2_O upward to the bottom of the continental plate without being exhausted (Fig. 4d). In the later stages of subduction (e.g. 95.4 Ma, Fig. 4d), some parts of the stagnant slab have mostly completed the dehydration process and retain only 0.2–0.3 wt% H_2_O. Notably, numerical modeling by Yang and Faccenda (2020) indicates that when water content in the mantle transition zone reaches 0.2-0.3%, localized melting and magmatic activity may be triggered, which is consistent with the observations in this study regarding the potential for deep dehydration to induce magmatic activity^87^.

**Fig. 5:**
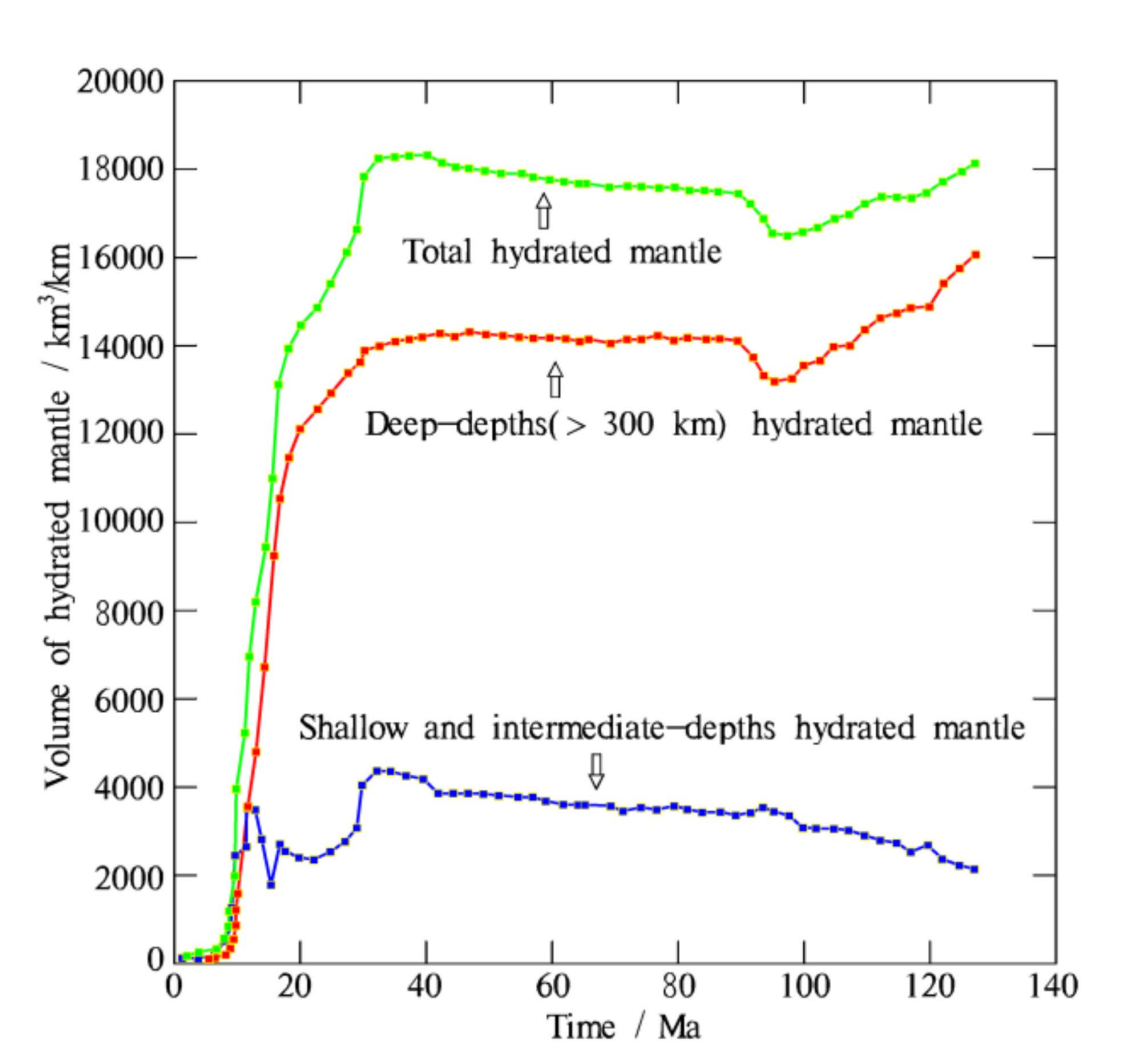
Volumes of hydrated mantle within a 0-140 Ma window for the reference model are measured in km^3^/km. Except for the initial period (<5 Ma), the total volume of hydrated mantle primarily comes from the deep-depths hydrated mantle.The map was generated using MATLAB (R2022a, MathWorks, https://www.mathworks.com).

The temporal evolution of the hydrated mantle volume (Fig. 5) shows that shortly after subduction initiation (<5 Ma), the mantle is hydrated by the sinking slab at shallow and intermediate depths (Fig. 4a). Around 10 Ma, the subducting slab bends at the ~ 660 km transition zone. As subduction continues, the stagnant slab progresses forward, leading to more of the slab lying on the transition zone and more deep mantle (>300 km) being hydrated by fluids released from the slab. The total volume of hydrated mantle primarily comes from the deep-depths hydrated mantle. Between 30 and 90 Ma, subduction slows down, and deep dehydration enters a stable period (Fig. 4b and c). After ~ 90 Ma, deep dehydration becomes active again as the surrounding hot mantle warms the slab sufficiently, leading to episodic subduction (Fig. 5). During this period, the majority of fluids in the stagnant slab are consumed by dehydration and partial melting (Fig. 4d).

### P-T conditions and deep magmatism

**Fig. 6:**
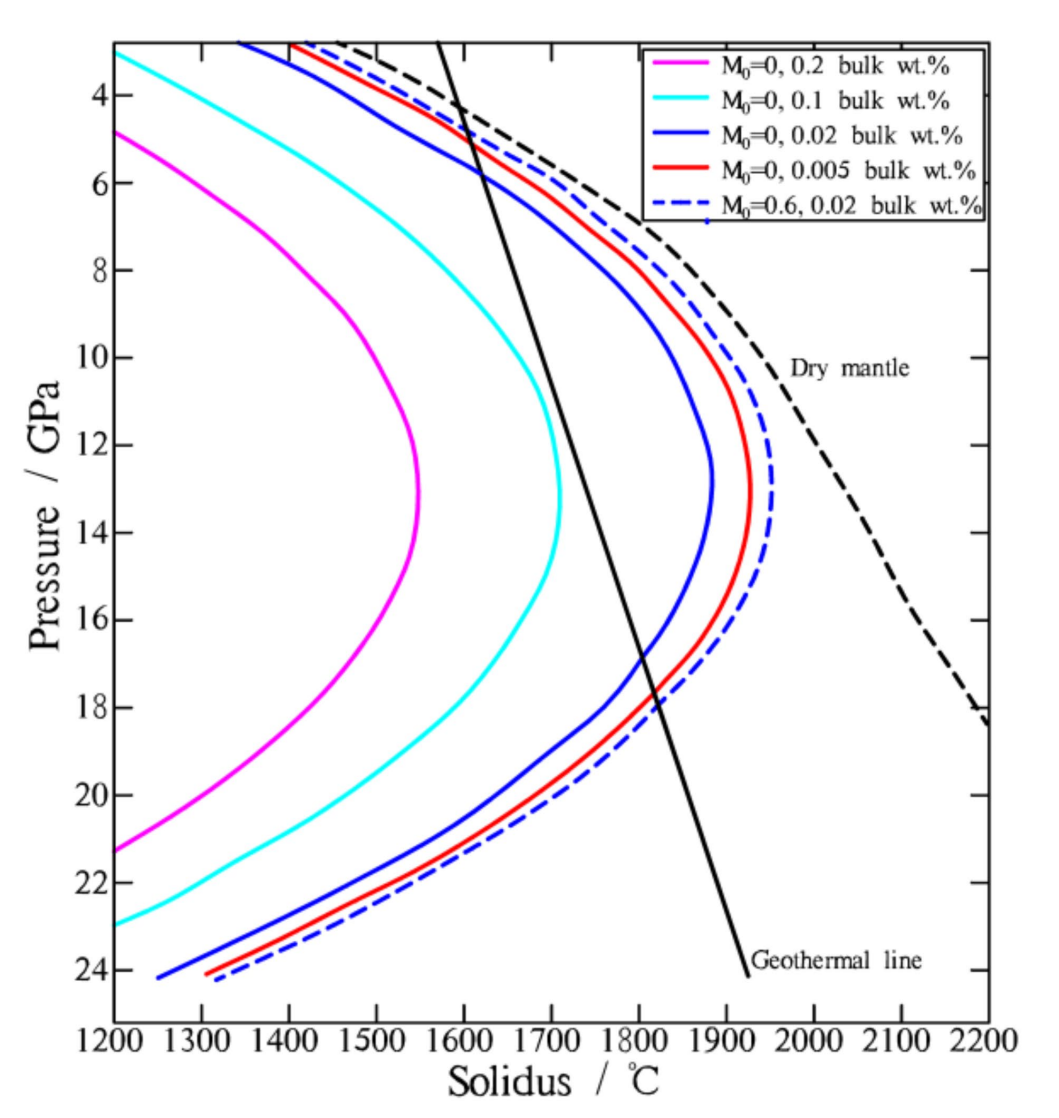
Solidus of peridotite in our models.$\:{\:\text{M}}_{0}$ represents the melt fraction ($\:{\:\text{M}}_{0}={\text{M}}_{\text{c}\text{p}\text{x}},\:\:\text{w}\text{h}\text{e}\text{n}\:{\text{T}}_{\text{s}\text{o}\text{l}\text{i}\text{d}\text{u}\text{s}}<\text{T}<{\text{T}}_{\text{c}\text{p}\text{x}-\text{o}\text{u}\text{t}}$ ,$\:\:{\:\text{M}}_{0}={\text{M}}_{\text{o}\text{p}\text{x}},\:\text{w}\text{h}\text{e}\text{n}\:{\text{T}}_{\text{c}\text{p}\text{x}-\text{o}\text{u}\text{t}}<\text{T}<{\text{T}}_{\text{l}\text{i}\text{q}\text{u}\text{i}\text{d}\text{u}\text{s}}$). The solidus decreases as the bulk water content varies from 0 to 0.2 wt%. The map was generated using MATLAB (R2022a, MathWorks, https://www.mathworks.com).

**Fig. 7:**
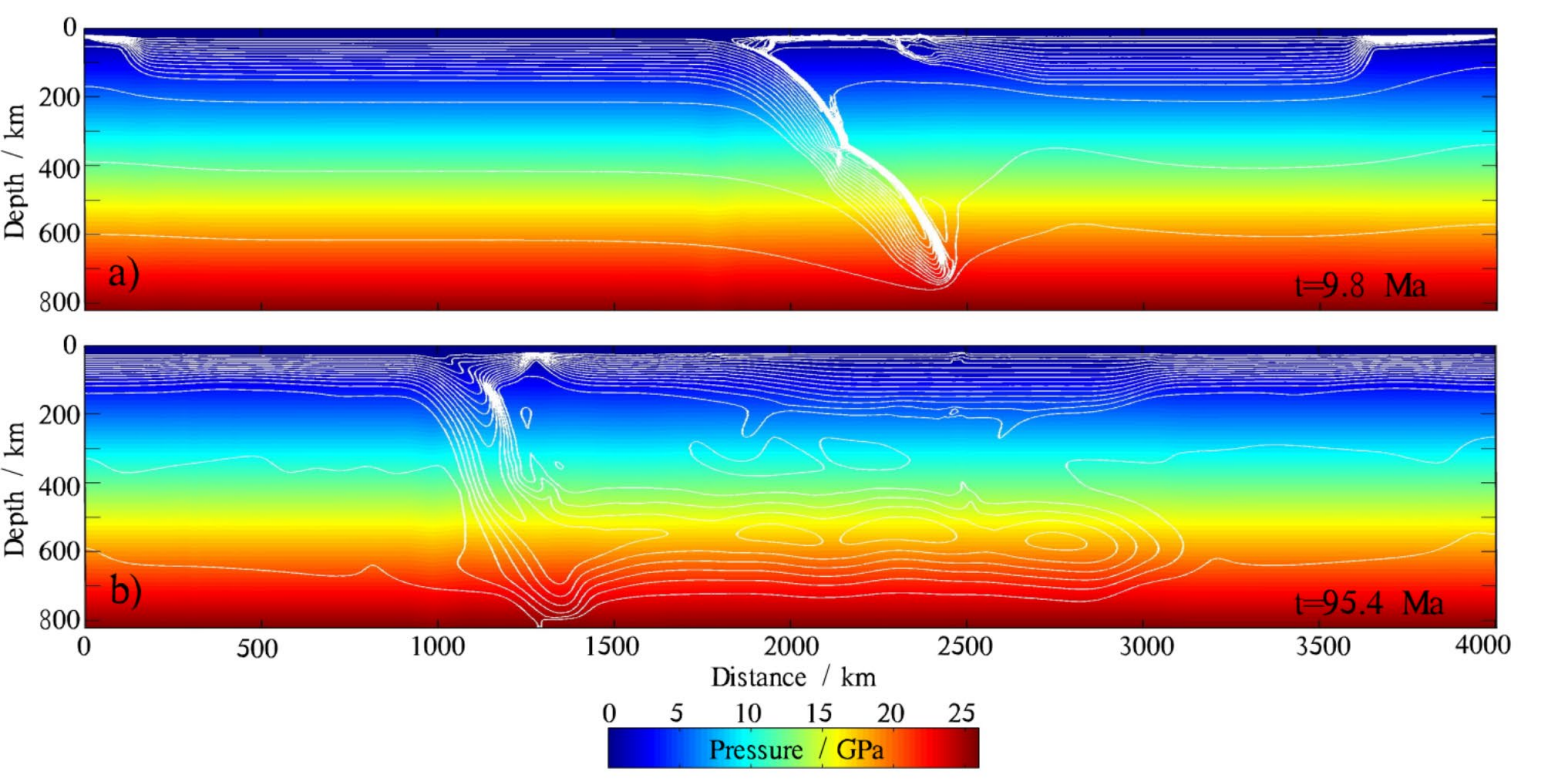
The P-T conditions of the reference model at 9.8 Ma and 95.4 Ma of subduction time. Isotherms are displayed as white lines with 100 ℃ increments, starting from 100 ℃.The map was generated using MATLAB (R2022a, MathWorks, https://www.mathworks.com).

The onset of mantle partial melting is controlled by its solidus curve, which is significantly influenced by the bulk water content. The solidus of the deep mantle can decrease by more than 1000 ℃ when the mantle transitions from dry to containing 0.2 wt% bulk water (Fig. 6). The melt fraction (*M*_0_) also influences the solidus of the mantle, though not significantly. The solidus of the mantle changes very little (no more than 100 ℃) when the melt fraction varies from 0 to 0.6 under the same hydrous conditions (Fig. 6). During subduction, the pressure field remains almost unchanged, while the temperature field varies by several hundred degrees Celsius in the deep mantle (Fig. 7). The temperature of the deep mantle decreases as heat is transferred to the subducting slab. This heat transfer warms the slab, causing it to release more fluid. The progressively increasing water released from the slab propagates upward into the surrounding mantle, lowering its solidus. The decrease in the solidus is much greater than the change in temperature. This is due to the increased partial melting in the deep mantle.

**Fig. 8:**
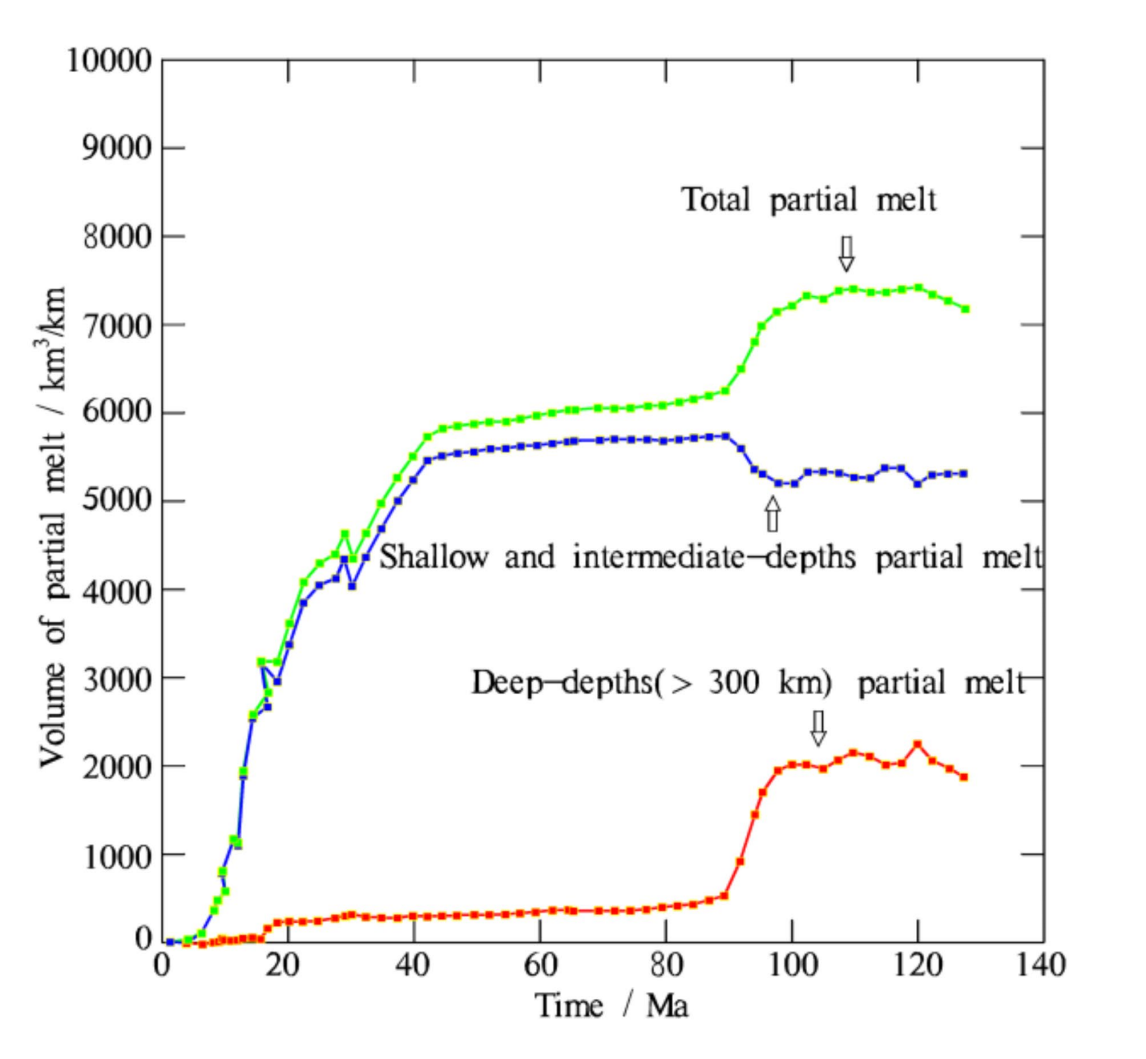
The volumes of partial melt within a 0-140 Ma window for the reference model are measured in km^3^/km. The majority of deep-depth partial melt is generated during the later stages of subduction (>90 Ma).The map was generated using MATLAB (R2022a, MathWorks, https://www.mathworks.com).

The temporal evolution of partial melt volume (Fig. 8) indicates that the generation of a large amount of partial melts in the deep mantle requires a long period of time. This time allows for sufficient water to be released from the subducting plate, ensuring that the solidus of the mantle is lowered to a sufficiently low level due to the increasing fluid content. Prior to this period, shallow and intermediate-depth partial melting dominates the total magma production. Thereafter, deep partial melting contributes increasingly to the total magma production (Fig. 8).

## Discussion

### Detached slab deep dehydration

**Fig. 9:**
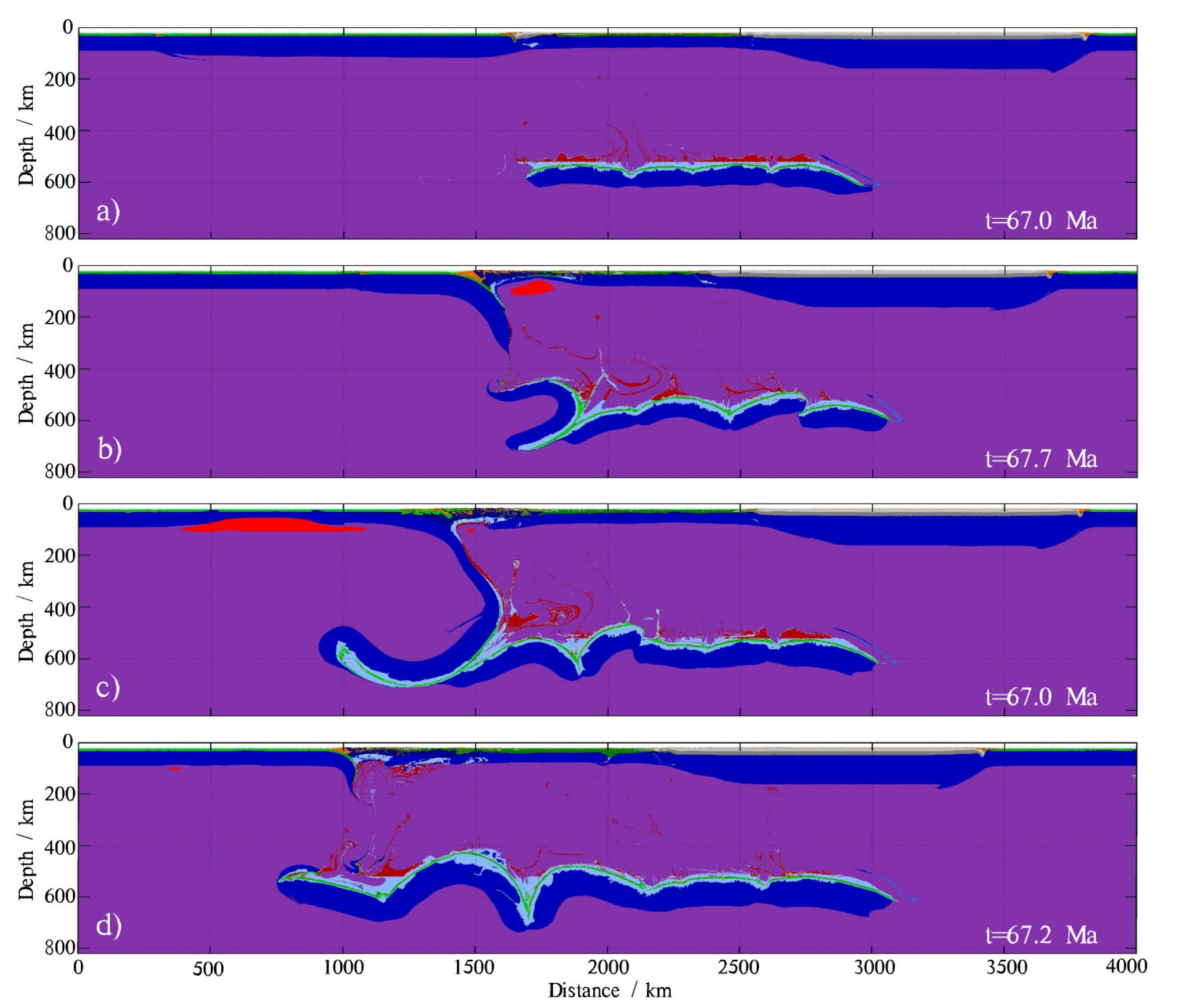
Color snapshots illustrate the dehydration of residual detached slabs in the deep mantle. (**a**) Slab breaks off at shallow depths during the early stage of subduction (<10 Ma). (**b**) Slab breaks off at intermediate depths (~ 300 km). (**c**) Slab breaks off at deep depths (~ 660 km). (**d**) Slab breaks off at shallow depths during the later stage of subduction (>50 Ma).The map was generated using MATLAB (R2022a, MathWorks, https://www.mathworks.com).

Slab detachments are frequently observed in subduction zones^88,89^. The detached slab lying on the ~ 660 km transition zone can also release fluids and trigger deep mantle partial melting under specific P-T conditions. These hydrous upwellings from the lower mantle, resulting from residual detached slab dehydration, can even trigger new subduction zones by hydrating the overriding lithosphere and developing a low-viscosity, dipping shear zone within it^15^. Our models simulate slab break-off from the surface down to the ~ 660 km transition zone. Various slab detachment depths have been proposed by several previous studies^90–94^. The timing of slab detachment varies from the very early stages of subduction (<10 Ma) to the later stages (>50 Ma). The age of the subducting slab is positively correlated with the time of slab detachment, meaning that the older the slab, the longer the detachment time. This relationship is due to the increase in slab thermal thickness, which prolongs the thermal relaxation time and thus delays the necking process^93,95^. Additionally, early detachment tends to occur at shallower depths. Slab detachment is a highly complex process governed by numerous parameters. In our experiments, not all later detachments occur at greater depths (Fig. 9d).

Figure 9 illustrates the deep dehydration of residual detached slabs in four models, each with different detachment depths and times. Shallow detachment (<300 km) causes the subducting slab to suddenly sink into the deep mantle. The upper portion of the subducting slab does not have enough time to dehydrate at shallow and intermediate depths before suddenly sinking. Consequently, more fluids are transported to the deep mantle along with the suddenly sinking upper portion of the detached subducting slab. This portion can release more fluids than others because it contains more water (Fig. 9a and d). The falling portions of intermediate and deep detachment (300–660 km) exhibit a similar volume distribution of slab dehydration as the models without slab detachment (Fig. 9b and c). Since the falling portions of intermediate and deep detachment have sufficient time for dehydration, the volume of water carried into the deep mantle by these falling portions does not change significantly. The duration of the slab detachment process is also a critical parameter that controls how much water can be carried into the deep mantle by the upper portion of the detached subducting slab. However, the duration of the slab detachment process is highly complex, as it is governed by numerous parameters. A systematic study of this topic is beyond the scope of this paper.

### Dehydration and solidus curves

In our models, the dehydration reaction is controlled using a dehydration curve proposed by Schmidt and Poli (SP1998)^23^. Faccenda et al^21^. suggested that a partially hydrated layer, where fluid concentration forms in the lithospheric mantle, develops more readily and at shallower depths (within the 450 ± 50 ℃ temperature range) using the dehydration curve proposed by Wunder and Schreyer^96^(WS1997) compared to SP1998. However, Faccenda et al^21^. suggested that under high confining pressures, the WS1997 dehydration curve has a very small and negative slope compared to SP1998. This is because the thickness of the Dehydration-Driven Zone (DHZ) is sensitive to the slab’s thermal structure rather than to the maximum temperature at which antigorite remains stable.

The solidus curve used in our models was proposed by Katz et al^63^., who provided a parameterization for melt fraction as a function of pressure, temperature, water content, and modal clinopyroxene (cpx), based on data from recent thermodynamic modeling and experimental studies. The parameterization represents a simplification of the complex natural system. For simplicity, it does not take into account factors such as the exhaustion of aluminous phases, the loss of orthopyroxene (opx) from the residue, and compositional variability. The solidus curve by Katz et al^63^. is applicable up to a maximum pressure of about 8 GPa (~ 250 km), while we have linearly extrapolated the curve to 24 GPa (~ 750 km). To enhance accuracy, the solidus curve should be refined using additional data from high-pressure and high-temperature experimental studies on peridotite melting and hydrous equilibria. If the solidus curve is refined, we can expect simulation results that are closer to natural systems.

### Evidences for deep slab dehydration

There is limited and indirect evidence supporting dehydration at depths greater than 300 km, but there is gradually increasing evidence supporting slab dehydration at such depths. Recently, a diamond containing ~ 1.5 wt% H_2_O in hydrous ringwoodite provided evidence that, at least locally, the mantle transition zone may be close to water saturation^97^.Consistent with the findings of Cerpa et al^98^. suggesting that only the coldest slabs can transport water into the mantle transition zone, our results also indicate that the deep dehydration process is relatively slow but can lead to large-scale mantle melting. Schmandt et al^99^. suggested that producing up to 1% melt through the dehydration melting of hydrous ringwoodite, which is viscously entrained into the lower mantle, is feasible. This conclusion is supported by evidence indicating that partial melting near the ~ 410 km discontinuity in a bulk peridotite system with 1 wt% H_2_O could result in approximately 5% partial melt at this depth^100,101^. At this depth, the partition coefficient of H_2_O between wadsleyite and olivine is at least 5:1102. Recent experiments have suggested that the partition coefficient of H_2_O between ringwoodite and silicate perovskite at the 660 km transition zone is as high as 15:1^102^. In our experiments, we observed deep slab dehydration and associated magmatism. This regime has been proposed to explain the origin of far-field intercontinental volcanism and magmatism in East Asia^16,21,34,103–105^. Van Der Lee et al^15^. suggested that deep slab dehydration could trigger subduction initiation along a passive margin. High Q^−1^ and low-velocity anomalies above the stagnating Pacific slab beneath East Asia may be caused by the dehydration of DHMS phases or the diffusion of water from an H_2_O-undersaturated slab at the 660 km transition zone^104^.

## Conclusion

Our experiments reveal the complete dynamic process of deep slab dehydration and magmatism, and demonstrate the possibility of slab dehydration at the 660 km transition zone. The volume of partial melts produced by slab dehydration at shallow to intermediate depths (<300 km) is approximately 2.5 times greater than that produced by deep slab dehydration (>300 km) at around 95 Ma (Fig. 8). In contrast, the volume of hydrated mantle at deep depths is three times greater than that at shallow to intermediate depths (Fig. 5). This implies that the dehydration reactions in the deep region is relatively weaker than in the shallow region. Generating significant dehydration-induced partial melts at deep depths requires a long period, as it takes time to achieve the appropriate P-T conditions for hydrous melting in the deep mantle. The progressively increasing amount of water released from the slab propagates upward into the surrounding mantle, lowering its solidus. This contributes to the increased amount of partial melting in the deep mantle. Plumes resulting from deep dehydration-induced magmatism can upwell and form magma chambers beneath far-field intercontinental plates.

## Data Availability

All data generated or analysed during the current study available from the corresponding author on reasonable request.
