# Author Correction: Numerical modeling the process of deep slab dehydration and magmatism

**DOI:** 10.1038/s41598-024-84095-8

**Published:** 2025-01-09

**Authors:** Hao Wu, Jiacheng Lei, Zeyu Jia, Jian Sheng, Yinan Zhu, Jian Wang

**Affiliations:** College of Transportation Engineering of Nanjing Tech, Nanjing Tech University, Nanjing, 211816 China

Correction to: *Scientific Reports* 10.1038/s41598-024-78193-w, published online 04 November 2024

The original version of this Article contained an error in the Affiliation.

Affiliation 1 was incorrectly given as ‘College of Transportation Science & Engineering, Nanjing Technology University, Zhongshan North Road 200, Nanjing, 180009, China’. The correct affiliation is listed below.

‘College of Transportation Engineering of Nanjing Tech, Nanjing Tech University, Nanjing 211816, China.’

The original Article has been corrected.

